# TFE3 fusion proteins drive *TFE3* rearranged renal cell carcinoma progression via PGC-1α-mediated fatty acid oxidation

**DOI:** 10.3389/fimmu.2026.1700983

**Published:** 2026-02-04

**Authors:** Fan Feng, Yanhao Xu, Zhenggen Deng, Xiang Dong, Guijuan Chen, Wenliang Ma, Dongmei Li, Weidong Gan

**Affiliations:** 1 Department of Urology, Nanjing Drum Tower Hospital, Affiliated Hospital of Medical School, Nanjing University, Nanjing, Jiangsu, China; 2 Department of Urology, The Affiliated Yantai Yuhuangding Hospital of Qingdao University, Yantai, China; 3 Robotic Minimally Invasive Surgery Center, Sichuan Provincial People’s Hospital, School of Medicine, University of Electronic Science and Technology of China, Chengdu, Sichuan, China; 4 State Key Laboratory of Analytical Chemistry for Life Science, Division of Anatomy and Histo-embryology, Medical School, Nanjing University, Nanjing, Jiangsu, China; 5 Jiangsu Key Laboratory of Molecular Medicine, Medical School, Nanjing University, Nanjing, Jiangsu, China; 6 Department of Thoracic Surgery, Nanjing Drum Tower Hospital, Affiliated Hospital of Medical School, Nanjing University, Nanjing, Jiangsu, China

**Keywords:** cancer progression, fatty acid metabolism, PGC1 α, renal cell carcinoma, TFE3

## Abstract

**Introduction:**

*TFE3* rearranged renal cell carcinoma (*TFE3* rRCC) is a distinct and aggressive subtype of RCC characterized by poor prognosis. While TFE3 fusion proteins are central to its pathogenesis, their specific roles in tumor progression, particularly regarding metabolic regulation, remain incompletely understood. This study investigates whether *TFE3* fusion proteins promote *TFE3* rRCC progression by regulating fatty acid oxidation (FAO).

**Methods:**

To elucidate the regulatory mechanisms, transcriptome sequencing, Western blotting, real-time quantitative PCR, dual-luciferase reporter assays, Chromatin Immunoprecipitation assays, and Seahorse XF96 analysis were employed to examine how TFE3 fusion proteins regulate the PGC-1α/PPARα/CPT1A axis and its impact on mitochondrial FAO in tumor cells. Additionally, bioinformatics analysis of publicly available TCGA data was conducted to assess the expression of *PGC1A* and *CPT1A* in various kidney cancer subtypes and their correlation with patient prognosis.

**Results:**

*TFE3* fusion proteins were found to transcriptionally upregulate PGC-1α, thereby increasing the tumor cells dependency on mitochondrial FAO. Mechanistically, PGC-1α co-activated PPARα to promote the expression of CPT1A, a rate-limiting enzyme in FAO. This TFE3/PGC-1α/CPT1A axis enhanced tumor cell proliferation, migration, and invasion. TCGA data analysis revealed that low expression levels of PGC1A and CPT1A in general kidney cancer are associated with poor patient prognosis. Conversely, in our specific *TFE3* rRCC cohort, high expression of PGC-1α and CPT1A correlated with poorer survival outcomes, highlighting their clinical significance.

**Conclusions:**

*TFE3* fusion proteins enhance FAO and drive *TFE3* rRCC progression via the PGC-1α/PPARα/CPT1A axis. Targeting CPT1A could inhibit tumor cell proliferation, suggesting that this pathway may serve as a potential therapeutic target for *TFE3* rRCC.

## 1 Introduction

*TFE3* rearranged renal cell carcinoma (*TFE3* rRCC) is a distinct and aggressive subtype of renal cell carcinoma (RCC) caused by translocations involving the *TFE3* gene on the X chromosome and other partner genes. This genetic alteration leads to the high expression of TFE3 fusion proteins (1). Clinical studies have consistently shown that RCCs with high expression of TFE3 fusion proteins are highly aggressive, exhibiting a high propensity for lymph node and distant metastasis, and are generally associated with poorer survival outcomes compared to common RCC subtypes (2, 3). The *TFE3* fusion genes typically retain the structural functional domains of the wild-type *TFE3* gene, resulting in translated protein with similar, yet often dysregulated, functions (4). Wild-type TFE3 protein is known to promote mitochondrial biogenesis and enhances mitochondrial oxidative phosphorylation by regulating mitochondrial-related genes (5). TFE3 fusion proteins, a defining feature of *TFE3* rRCC, also play a crucial role in maintaining mitochondrial homeostasis. For instance, the PRCC-TFE3 fusion protein has been shown to mediate parkin-dependent mitophagy, thereby regulating mitochondrial quality control and promoting tumor progression (6). Given the critical involvement of TFE3 fusion proteins in mitochondrial metabolism and the current lack of highly effective therapeutic strategies for *TFE3* rRCC, an in-depth investigation into the molecular mechanisms underlying tumor progression, particularly metabolic reprogramming, is warranted. Our team’s previous metabolomic and functional studies have confirmed that *TFE3* rRCC prefers mitochondrial respiration over glycolysis (7). In this study, through mRNA sequencing combined with bioinformatics analysis, we identified that peroxisome proliferator-activated receptor γ coactivator 1 alpha (*PGC1A*) as a potentially novel pathogenic gene in *TFE3* rRCC.

*PGC1A* is a central molecule through which wild-type TFE3 regulates mitochondrial function; specifically, wild-type TFE3 promotes mitochondrial biogenesis and enhances mitochondrial oxidative phosphorylation by regulating *PGC1A* (8, 9). *PGC1A* encodes PGC-1α, a member of the PGC1 family of transcriptional coactivators. Its primary function involves modifying chromatin, unwinding the DNA double helix, and recruiting RNA polymerase, thereby assisting transcription factors in their regulatory roles (10). PGC-1α acts synergistically with various transcription factors involved in mitochondrial metabolic processes, such as nuclear respiratory factors and peroxisome proliferator-activated receptors (PPARs) (11, 12). Our previous work demonstrated that TFE3 fusion proteins promote lipophagy to increase the intracellular pool of free fatty acids (FAs) (13). However, the downstream mechanisms by which these FAs are utilized to fuel tumor progression remain largely unexplored. In this study, through RNA sequencing, we identified that *PGC1A* might be a key downstream effector in this process. Therefore, it is imperative to clarify the pathogenic role of *PGC1A* in the initiation and progression of *TFE3* rRCC, as well as whether it is regulated by TFE3 fusion proteins.

In lipid metabolism, FAs generated from the degradation of intracellular lipid droplets can activate PPARs to promote lipid breakdown or be transported to mitochondria, where they are converted into acetyl-CoA for energy production (14–16). The PPARs family consists of three isoforms: PPARα, PPARβ/δ, and PPARγ. These receptors play crucial roles in lipid metabolism, glucose homeostasis, and immune regulation by modulating processes such as fatty acid oxidation (FAO), energy metabolism, adipogenesis, and inflammatory responses (17). Carnitine palmitoyltransferase 1 (CPT1), located on the outer mitochondrial membrane, transports long-chain FAs into mitochondria for oxidation and acts as the rate-limiting enzyme in this process, with CPT1A and CPT1B being the main isoforms expressed in humans (18). Studies have shown that PGC-1α collaborates with the transcription factor PPARα to regulate the expression of CPT1 and other mitochondrial FAO enzymes, thereby enhancing the rate of mitochondrial FAO (19). However, the mechanism by which PGC-1α regulates FAO in *TFE3* rRCC remains unexplored.

In this study, we investigated the role of *PGC1A* in TFE3 rRCC, confirming *its* oncogenic potential and its association with patient prognosis. Our findings indicated that TFE3 fusion proteins transcriptionally upregulated *PGC1A*. PGC-1α, in turn, co-activated PPARα to upregulate *CPT1A*, which significantly enhanced FAO. Furthermore, we demonstrate that targeting CPT1A inhibits tumor growth, thereby offering a potential therapeutic target for *TFE3* rRCC.

## 2 Materials and methods

### 2.1 Bioinformatics analysis

RNA-sequencing data were retrieved from The Cancer Genome Atlas (TCGA, https://portal.gdc.cancer.gov/) and a Gene Expression Omnibus (GEO, https://www.ncbi.nlm.nih.gov/geo/) dataset (GSE188885) (20). Clinical data was obtained from TCGA for patients with Kidney Renal Clear Cell Carcinoma (KIRC), Kidney Renal Papillary Cell Carcinoma (KIRP), and Kidney Chromophobe (KICH). Our own mRNA sequencing of *TFE3* rRCC cell line samples was conducted by Wuhan Kangce Technology. VENNY 2.1 was used to generate Venn diagrams for visualizing overlapping differentially expressed genes (DEGs) from GSE188885 and our mRNA sequencing results. The R package “pheatmap” was utilized to display the expression levels of candidate gene sets as heatmaps. Gene Ontology (GO) functional annotations and Kyoto Encyclopedia of Genes and Genomes (KEGG) pathway analyses were performed to identify the top 100 up-regulated and down-regulated DEGs. Kaplan-Meier survival analyses were applied to TCGA samples of KIRC and KIRP to explore the prognostic significance of relevant genes, including analyses of overall survival (OS), disease-specific survival (DSS), and progression-free interval (PFI). The diagnostic performance of *PGC1A* was evaluated by constructing receiver operating characteristic (ROC) curves and calculating the area under the curve (AUC) using the R package “pROC”. Pearson’s correlation test was employed to examine the relationship between MiT transcription factors and *PGC1A*, with the results visualized as scatter plots. Based on Spearman’s correlation test, the R package “ggplot2” was used to create a preranked list of DEGs sorted by their relationship with *PGC1A*. Protein-protein interactions were analyzed using STRING.

### 2.2 Clinical samples collection

Thirty paraffin-embedded tissue specimens of human *TFE3* rRCC were collected from Nanjing Drum Tower Hospital. These samples were derived from patients with complete clinical records, and their diagnoses were verified by TFE3 immunohistochemistry (IHC), fluorescence *in situ* hybridization (FISH), and transcriptome sequencing. The study cohort included 13 male and 17 female patients, with a median age of 38 years (ranging from 22 to 71 years). Additionally, thirty paired tumor tissue samples were gathered from clear cell renal cell carcinoma (ccRCC) patients, selected based on tumor size and pathological stage, with the median age of these patients being 59 years. All patients provided written informed consent, and the study protocol was approved by the Institutional Review Board of Affiliated Drum Tower Hospital, Medical School of Nanjing University.

### 2.3 Cell culture and transfection

HK-2, HEK-293T, ACHN and 786-O cell lines were obtained from the ATCC cell bank. The *TFE3* rRCC cell lines UOK120 (*PRCC*-*TFE3* fusion gene) and UOK109 (*NONO*-*TFE3* fusion gene) were kindly provided by the National Cancer Institute of the National Institutes of Health. All cells were cultured in 90% Dulbecco’s Modified Eagle Medium (DMEM, Gibco, 10569010), supplemented with 10% fetal bovine serum (FBS, Gibco, 16140063) and 1% penicillin/streptomycin (Invitrogen, 15070063). Cell lines were incubated at 37°C with 5% CO_2_. For all experiments, cells were used between passages 5 and 10 to ensure consistency in cellular behavior and minimize genetic drift. To generate stable cell populations with *TFE3, PGC1A and CPT1A* knockdown or overexpression, cells were transfected with the corresponding plasmids (**Supplementary Table S1**). To generate lentiviruses, the psPAX2 packaging plasmid, pMD2.G envelope plasmid, and the transfer plasmid were co-transfected into HEK293T cells. Viral supernatants were collected 48 and 72 hours post-transfection.

### 2.4 Real-time PCR

Total RNA was extracted using TRIzol reagent (Vazyme, R401) and subsequently reverse-transcribed using the HiScript II Reverse Transcriptase Master Mix Kit (Vazyme, R201) following the manufacturer’s instructions. All primers (**Supplementary Table S2**) were synthesized by Tsingke Biotechnology. PCR amplification was performed using SYBR Green (Vazyme, Q711), and the reaction was quantified using an ABI ViiA 7 Q-PCR System (Applied Biosystems). The relative expression levels were calculated using the 2 ^–(ΔΔCt)^ method and normalized to the expression of 18S rRNA.

### 2.5 Western blot and co-immunoprecipitation

Total protein was extracted from control and treated cells using RIPA buffer (Beyotime, P0013C) supplemented with protease inhibitors (MCE, HYK0010) and phosphatase inhibitors (MCE, HY-K0022), while keeping the samples on ice. Protein concentration was determined using a BCA Protein Assay Kit (Vazyme, E112-01). Equal amounts of protein were resolved by 10% SDS-PAGE and transferred to a nitrocellulose membrane for 75 minutes at 100 V. The membranes were blocked with tris-buffered saline containing 0.1% Tween 20 (TBS-T) and 5% skim milk, followed by overnight incubation with primary antibodies (**Supplementary Table S3**) at 4°C. After washing, membranes were incubated with HRP-conjugated secondary antibodies (**Supplementary Table S3**) for 1 hour at room temperature. Protein bands were visualized using ECL solution (Millipore) and quantified with ImageJ software. Co-IP was performed using a Pierce Magnetic Co-IP Kit (Thermo Fisher Scientific, 88804) according to the manufacturer’s protocol. The supernatant was collected for subsequent Western blot analysis.

### 2.6 IHC and immunofluorescence

Paraffin-embedded tissue specimens were sectioned into 4-micron slices. After routine deparaffinization, rehydration, and blocking procedures, the sections were incubated with primary antibodies targeting the proteins of interest (**Supplementary Table S3**). On the following day, the sections were treated with secondary antibodies (**Supplementary Table S3**), followed by staining with diaminobenzidine (Servicebio, G1216-2) and hematoxylin (Servicebio, G1004). The staining intensity was quantified with Image J software. For IF, cells cultured on glass-bottom dishes were sequentially fixed with 4% paraformaldehyde, permeabilized with 0.1% Triton X-100, and blocked with 5% BSA. The cells were then incubated overnight with the appropriate primary antibodies at 4°C, followed by a 1-hour incubation using secondary antibodies (**Supplementary Table S3**) at room temperature. The glass-bottom dishes were mounted with 4’,6-diamidino-2-phenylindole (DAPI, Beyotime, P0131) for nuclear staining. Fluorescent images were acquired and analyzed using a confocal microscope (Olympus FV3000).

### 2.7 Cellular growth assays

Cell growth was evaluated using CCK-8 assays, EdU staining, and colony formation assays. For the CCK-8 assay, cells were seeded in 96-well plates, and absorbance at 450 nm was measured at 24, 48, 72, 96, and 120 hours following the addition of CCK-8 solution (Beyotime, C0037). In the EdU assay, EdU staining was performed using a BeyoClick EdU-594 kit (Beyotime, C0078S). Before EdU staining, UOK109 cells were incubated with EdU for 3 hours, while UOK120 cells were incubated for 6 hours. The positive rate was calculated as the percentage of EdU-positive cells relative to the total cell count in randomly selected fields. For colony formation assay, cells were seeded in Matrigel-coated plates (Green Micro & Nano, GMN-A-CTA-001) and cultured for 1–2 weeks. Colonies were then fixed, stained with 1% crystal violet solution, and counted and photographed.

### 2.8 Cell migration and invasion assays

Cell migration and invasion were assessed via Transwell chambers (Corning 3415). For migration, cells treated with 1% FBS for 24h were trypsinized, washed, resuspended in DMEM (1×10^6^ cells/mL), with 100μL added to upper chambers and 600μL complete medium to lower chambers. After 36-48h, migrated cells were fixed (4% paraformaldehyde) and stained (1% crystal violet). Invasion assays utilized upper chambers precoated with 4°C-liquid Matrigel (Green Micro & Nano, GMN-A-CTA-001) diluted 1:8 in DMEM, followed by the same steps. Both assays were performed in triplicate. The migrated cells were imaged and counted under a microscope (Nikon 50i; 3–5 fields averaged).

### 2.9 Seahorse XFe96 long-chain FAO stress test

Following the manufacturer’s instructions (Agilent, 103672-100), cells were seeded into a 96-well plate, with 1×10^4^ UOK109 cells or 8×10^3^ UOK120 cells per well. Then, cells were cultured to a confluency of 80-90% overnight. The XF calibration solution was incubated, and the probe plate was hydrated overnight. On the following day, the probes were hydrated, and the detection medium was prepared. After washing the cells, they were placed in an incubator for one hour. Appropriate concentrations of Etomoxir (Eto, 40μM), Oligomycin (15μM), Carbonyl cyanide p-trifluoromethoxyphenylhydrazone (FCCP, 1μM for UOK109, 2μM for UOK120), and Rotenone/Antimycin A (Rot/AA, 1μM) working solutions were prepared and added to the wells of the probe plate. Parameters were set according to the experimental design, and the assay was run on a Seahorse XFe96 Analyzer. Data were exported after the procedure finished. The concentration of protein in each well was detected using BCA method for normalization.

### 2.10 Flow cytometry analysis

Cells were washed, collected, and resuspended before staining with the Annexin V-PE/7-AAD Apoptosis Detection Kit (Vazyme, A213-01). After incubating at room temperature in the dark for 10 minutes, additional Binding Buffer was added, and samples were analyzed within 1 hour using a CYTOFLEX flow cytometer (Beckman Coulter). Negative, Annexin V-PE single-positive, and 7-AAD single-positive controls were set up to ensure accurate detection.

### 2.11 Luciferase reporter assay

The *PGC1A* promoter was inserted into the pGL3-Basic vector, and HEK-293T cells were transfected with the *PGC1A* promoter plasmid (**Supplementary Table S2**), which contains the firefly luciferase gene, using Lipofectamine 2000. The PRL-TK plasmid (Promega, E2241) was co-transfected at a 100:1 ratio as an internal control. After 48 hours of transfection, cells were lysed using the Dual-Luciferase Reporter Assay Kit (Vazyme, DL101), and luciferase activity was measured using a GloMaxTM 96 Microplate Luminometer (Promega).

### 2.12 Chromatin immunoprecipitation

The ChIP assays were conducted using the Pierce Agarose ChIP Kit (Thermo Fisher Scientific, 26156) in accordance with the provided protocol. DNA levels were quantified by qRT-PCR, and the specific primers used for ChIP are listed in **Supplementary Table S4**.

### 2.13 Mouse model

All animal experiments performed in this study were approved by The Animal Care and Use Committee of Nanjing University (Approval Number: D2402106). Initially, A498 cells were transfected with pCDH-DsRed/pCDH-NONO-TFE3, pCDH-DsRed/pCDH-PRCC-TFE3. Following transfection, cells were selected with puromycin until all untransfected cells were eliminated. When the cells reached a sufficient quantity, 3×10^6^ cells in each group were resuspended in 50μL of EMEM medium and 100μL of Matrigel, and then subcutaneously injected into the posterior axillary region of the forelimbs of nude mice (150μL per mouse). Ten days after tumor implantation, daily intraperitoneal injection of Eto at a dose of 40 mg per kilogram of body weight was initiated. After 3 weeks of Eto administration, the mice were euthanized humanely. The subcutaneous tumors were excised, measured for volume and weight, and preserved for subsequent studies, including H&E staining and IHC analysis.

### 2.14 Statistical analysis

Data are presented as mean ± standard deviation (mean ± S.D.) from independent experiments. Comparisons between two independent samples were performed using the Student’s t-test, while one-way ANOVA was used for comparisons among multiple groups. A *P*-value < 0.05 was considered statistically significant. Linear regression analysis was employed to assess the correlation between the expression levels of different molecules, with the correlation coefficient represented by R². Kaplan-Meier survival analysis was used to evaluate patient survival outcomes, and the Log-rank test was applied to determine differences in survival prognosis. Statistical analyses were performed using GraphPad Prism 8.0.1, and figures were generated using Adobe Photoshop CC2023 and Adobe Illustrator CC2023.

## 3 Results

### 3.1 *PGC1A* is a potential downstream target gene of TFE3 fusion proteins and correlates with RCC prognosis

**Figure 1:**
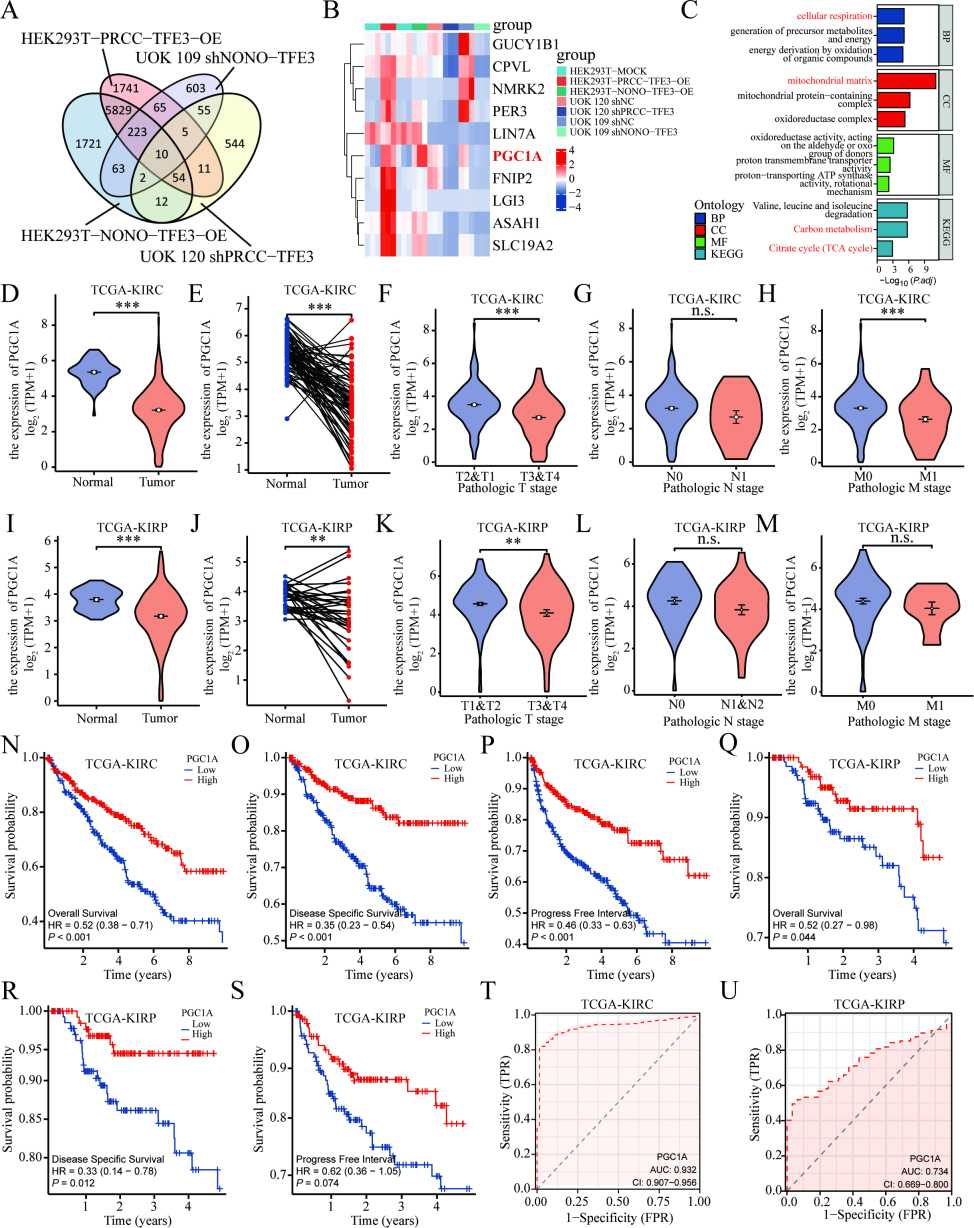
Poor prognosis in RCC patients with low *PGC1A* expression. **(A)** Venn diagram illustrating the intersection of transcriptome sequencing results from *TFE3* fusion gene knockdown and overexpression. **(B)** Heatmap showing the mRNA expression levels of candidate gene sets. **(C)** GO and KEGG pathway analyses of the gene set obtained from the single-gene differential analysis of *PGC1A*. **(D, E)** TCGA database analysis of the differential expression of *PGC1A* between KIRC and normal tissues. **(F–H)** TCGA database analysis of the correlation between *PGC1A* expression levels and pathological TNM staging in KIRC tumor tissues. **(I, J)** TCGA database analysis of the differential expression of *PGC1A* between KIRP and normal tissues. **(K–M)** TCGA database analysis of the correlation between *PGC1A* expression levels and pathological TNM staging in KIRP tumor tissues. **(N–S)** Survival analysis of the correlation between *PGC1A* expression levels in KIRC and KIRP tumor tissues and patient prognosis. **(T, U)** TCGA database analysis of the ROC curves for *PGC1A* in predicting prognosis for KIRC and KIRP tumor patients. **P < 0.01; ***P < 0.001.

To investigate how the TFE3 fusion proteins affected the development process of *TFE3* rRCC, mRNA sequencing was performed on cells with knockdown or overexpression of *TFE3* fusion genes compared to control group (GSE269083). A four-way Venn diagram identified ten candidate genes whose expression was altered in response to TFE3 fusion protein modulation (**Figure 1A**). These ten DEGs included *GUCY1B1*, *CPVL*, *NMRK2*, *PER3*, *LIN7A*, *PGC1A*, *FNIP2*, *LGI3*, *ASAH1*, and *SLC19A* (**Figure 1B**). Notably, the expression pattern of *PGC1A* in response to either knockdown or overexpression of *TFE3* fusion genes mirrored that of the *TFE3* fusion genes (**Figures 1A, B**). To further explore the cellular processes and pathways involving *PGC1A*, a single-gene differential expression analysis was conducted using KIRC transcriptome data from TCGA, followed by GO analysis of the identified gene set. The results indicated that the *PGC1A* differential expression gene set was enriched in biological processes related to cellular respiration and energy metabolism (**Figure 1C**). KEGG pathway analysis also revealed enrichment in both carbon metabolism and the mitochondrial tricarboxylic acid cycle (**Figure 1C**).

To investigate the broader role of *PGC1A* in renal malignancies, bioinformatics analysis of the TCGA database was conducted to assess the expression levels of *PGC1A* in common RCC subtypes, including KIRC and KIRP. Compared to normal tissues, *PGC1A* expression was found to be reduced in both KIRC and KIRP tissues (**Figures 1D, E, I, J**). Furthermore, *PGC1A* expression levels decreased in correlation with higher tumor pathological T staging in both KIRC and KIRP (**Figures 1F, K**). In KIRC, *PGC1A* expression was also associated with tumor M staging, whereas no such correlation was observed in KIRP (**Figures 1H, M**). Although *PGC1A* expression tended to be lower in high N-stage tumor tissues, no statistically significant differences were observed between KIRC and KIRP (**Figures 1G, L**). Kaplan-Meier survival analysis showed that KIRC patients with low *PGC1A* expression had significantly poorer OS, DSS, and PFI compared to those with high expression (**Figures 1N–P**). Similarly, in KIRP, patients with low *PGC1A* expression exhibited significantly lower OS and DSS than those with high expression (**Figures 1Q–S**). ROC curve analysis indicated that *PGC1A* may serve as a diagnostic biomarker for KIRC and KIRP, with AUC values of 0.932 and 0.734, respectively, reflecting high sensitivity and specificity (**Figures 1T, U**). The wild-type *TFE3* gene, along with *MITF, TFEB, and TFEC*, belongs to the MiT transcription factor family. Correlation analysis using the TCGA database revealed a positive correlation between *MITF, TFEB, TFEC*, and *TFE3* expression levels with *PGC1A* expression in KIRC, KIRP, and KICH tumor tissues (**Supplementary Figures S1A–L**). These findings suggest that *PGC1A* might be a potential downstream target gene of *TFE3* fusion proteins, and that low expression of *PGC1A* is associated with poor prognosis in KIRC and KIRP patients within the TCGA cohort.

### 3.2 TFE3 fusion proteins transcriptionally upregulate *PGC1A* without affecting its subcellular localization

**Figure 2:**
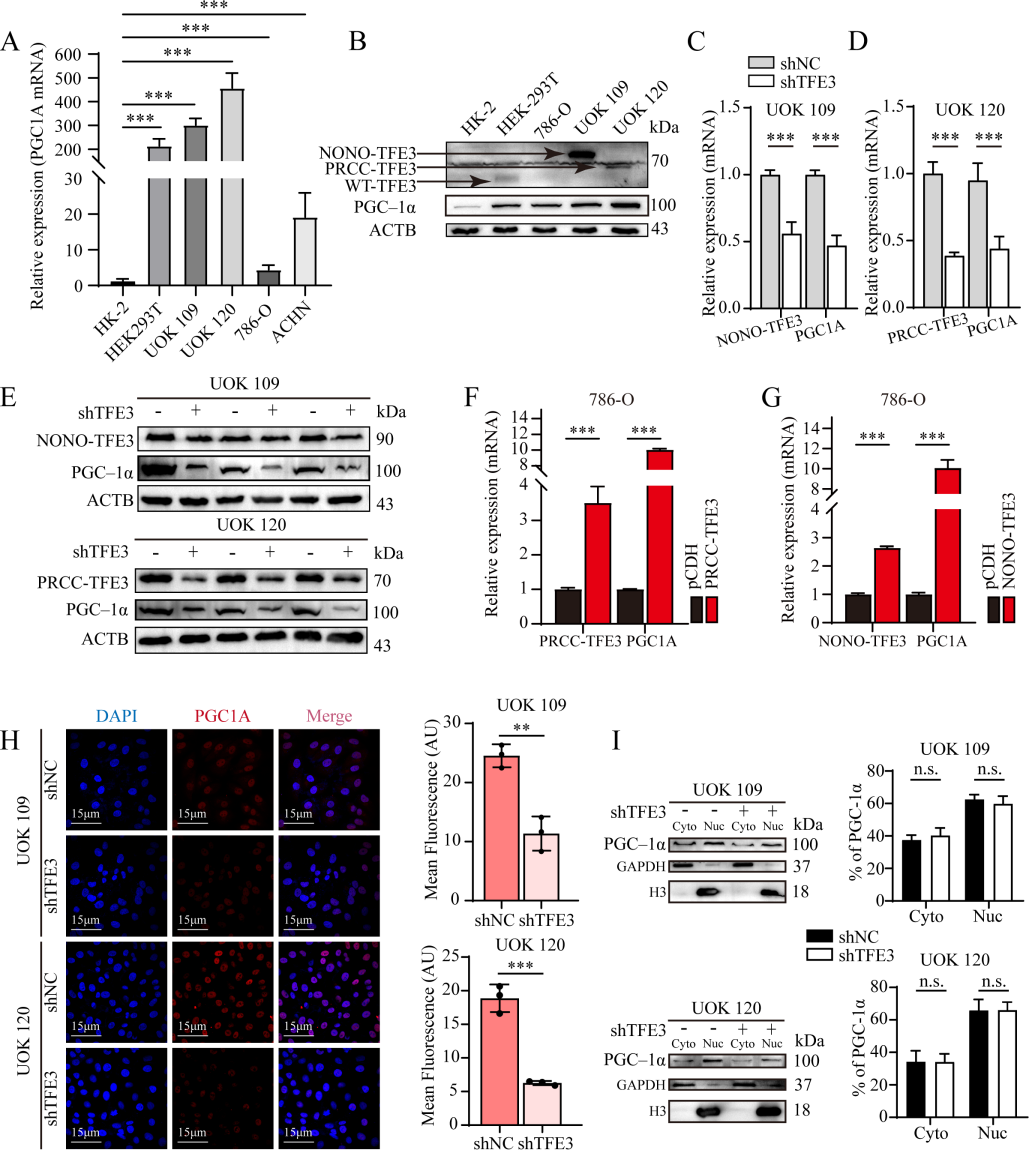
Silencing the *TFE3* fusion gene significantly downregulates PGC-1α expression without affecting its subcellular localization. **(A)** Real-time PCR analysis of *PGC1A* expression in RCC cell lines and normal renal tubular epithelial cell lines. **(B)** Western blot analysis of PGC-1α expression in RCC cell lines and normal renal tubular epithelial cell lines. **(C–E)** Western blot and Real-time PCR analysis of *TFE3* fusion gene and *PGC1A* protein and mRNA expression levels in UOK109 and UOK120 cells. **(F, G)** Real-time PCR analysis of *PGC1A* expression in 786-O cells overexpressing the *TFE3* fusion gene. **(H)** IF of PGC-1α subcellular localization in UOK109 and UOK120 cells in the shNC and sh*TFE3* groups. **(I)** Cytoplasmic-nuclear fractionation analysis of PGC-1α distribution in UOK109 and UOK120 cells in the shNC and sh*TFE3* groups. **P < 0.01; ***P < 0.001.

To reveal the regulatory mechanism of TFE3 fusion proteins on *PGC1A*, protein and mRNA levels of *PGC1A* were assessed in various cell lines using Western blotting and qRT-PCR. The results showed that *PGC1A* mRNA expression was significantly higher in UOK109 and UOK120 cells compared to HK-2 and 786-O cells (**Figure 2A**). A similar trend was observed for PGC-1α expression levels (**Figure 2B**). Following knockdown of the *TFE3* fusion gene in tumor cells, qRT-PCR analysis showed a significant reduction in *PGC1A* expression (**Figures 2C, D**). Consistent with these findings, PGC-1α protein levels were also notably decreased following *TFE3* fusion gene knockdown (**Figure 2E**). In contrast, when the *TFE3* fusion gene was transfected into 786-O cells, total cellular RNA was extracted, and q-RT-PCR analysis revealed that overexpression of the *TFE3* fusion gene significantly upregulated *PGC1A* expression compared to the control group (**Figures 2F, G**). Given that PGC-1α is a transcriptional co-activator primarily active in the nucleus, confocal microscopy was employed to assess the subcellular localization of PGC-1α to determine whether TFE3 fusion proteins influence its intracellular distribution. The results indicated that knockdown of the *TFE3* fusion gene significantly reduced PGC-1α expression in the nucleus (**Figure 2H**). Moreover, when cytoplasmic and nuclear protein fractions were separated, it was found that PGC-1α was predominantly localized in the nucleus, and knockdown of the *TFE3* fusion gene did not affect the subcellular distribution ratio of PGC-1α (**Figure 2I**).

**Figure 3:**
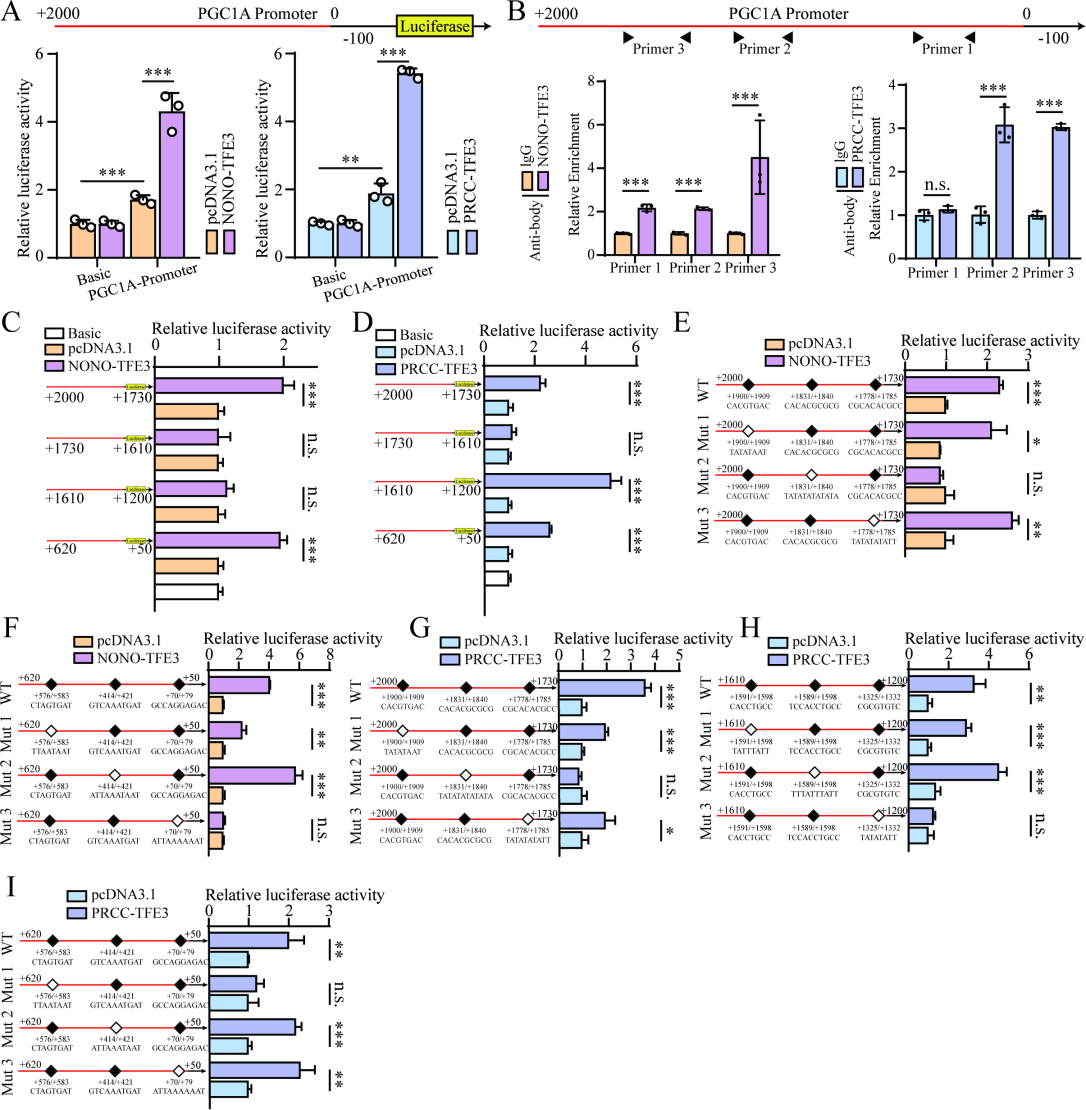
*TFE3* fusion proteins enhance *PGC1A* transcription. **(A)** Dual-luciferase reporter assay assessing the regulatory effect of NONO-*TFE3* and PRCC-*TFE3* fusion proteins on the *PGC1A* promoter region. **(B)** ChIP analysis of the binding of endogenous NONO-*TFE3* and PRCC-*TFE3* fusion proteins to the *PGC1A* promoter region. **(C, D)** Investigation of the actual binding sites of NONO-*TFE3* and PRCC-*TFE3* fusion proteins on *PGC1A* using truncated plasmids. **(E, F)** Identification of the binding sites of NONO-*TFE3* fusion proteins on *PGC1A* via site-directed mutagenesis plasmids. **(G–I)** Identification of the binding sites of PRCC-*TFE3* fusion proteins on *PGC1A* using site-directed mutagenesis plasmids. *P < 0.05; **P < 0.01; ***P < 0.001.

To verify whether TFE3 fusion proteins transcriptionally regulated *PGC1A* expression, the *PGC1A* promoter sequence was inserted into the pGL3-Basic plasmid to construct a luciferase reporter plasmid. This plasmid, along with the *PRCC-TFE3* or *NONO-TFE3* overexpression plasmids, was co-transfected into HEK-293T cells to assess transcriptional activity. Dual-luciferase reporter assays revealed that both PRCC-TFE3 and NONO-TFE3 fusion proteins significantly enhanced luciferase activity compared to the control group containing an empty plasmid (**Figure 3A**). To further validate whether TFE3 fusion proteins directly regulate *PGC1A* transcription, ChIP assays demonstrated that both NONO-TFE3 and PRCC-TFE3 fusion proteins directly bind to the *PGC1A* promoter region in UOK109 and UOK120 cells (**Figure 3B**). Next, to identify the specific binding sites of TFE3 fusion proteins on the *PGC1A* promoter, the *PGC1A* promoter sequence was truncated into several fragments (+2000 bp to +1730 bp, +1730 bp to +1610 bp, +1610 bp to +1200 bp, +620 bp to +50 bp), which were then inserted into the pGL3-Basic plasmid to construct luciferase reporter plasmids. These truncated plasmids were co-transfected with *PRCC-TFE3* or *NONO-TFE3* overexpression plasmids into HEK-293T cells to examine transcriptional activity. Dual-luciferase reporter assays showed that, compared to the control group with an empty plasmid, NONO-TFE3 fusion protein did not affect transcription in the +1730 bp to +1610 bp and +1610 bp to +1200 bp regions, but it enhanced transcription in the other truncated sequences. This suggested that the NONO-TFE3 fusion protein regulated *PGC1A* transcription within the +2000 bp to +1730 bp and +620 bp to +50 bp promoter regions (**Figure 3C**). Similarly, with the exception of the +1730 bp to +1610 bp region, transcription in the other truncated sequences was enhanced by PRCC-TFE3 fusion protein, indicating that PRCC-TFE3 regulates *PGC1A* transcription in the +2000 bp to +1730 bp, +1610 bp to +1200 bp, and +620 bp to +50 bp regions (**Figure 3D**).

Based on JASPAR (http://jaspar.genereg.net/) online analysis, potential binding sites for the NONO-TFE3 fusion protein were identified in the +2000 bp to +1730 bp and +620 bp to +50 bp regions. These sites were individually mutated, and the normal or mutated plasmids were co-transfected with the *NONO-TFE3* overexpression plasmid into HEK-293T cells. Dual-luciferase reporter assays indicated that the transcriptional activity of the mutated sequences containing +1840 bp to +1831 bp and +79 bp to +70 bp was not affected by NONO-TFE3 fusion protein, suggesting that the actual binding sites of NONO-TFE3 on the *PGC1A* promoter were located between +1840 bp to +1831 bp and +79 bp to +70 bp (**Figures 3E, F**). Similarly, the potential binding sites for PRCC-TFE3 fusion protein in the +2000 bp to +1730 bp, +1610 bp to +1200 bp, and +620 bp to +50 bp regions were mutated. Dual-luciferase reporter assays showed that the transcriptional activity of the mutated sequences containing +1840 bp to +1831 bp, +1332 bp to +1325 bp, and +583 bp to +576 bp was not influenced by PRCC-TFE3 fusion protein, indicating that the actual binding sites of *PRCC-TFE3* on the *PGC1A* promoter were between +1840 bp to +1831 bp, +1332 bp to +1325 bp, and +583 bp to +576 bp (**Figures 3G–I**). These results collectively suggested that *TFE3* fusion proteins promote transcription by directly binding to the *PGC1A* promoter region.

### 3.3 High expression of PGC-1α enhanced FAO and promoted *TFE3* rRCC progression

**Figure 4:**
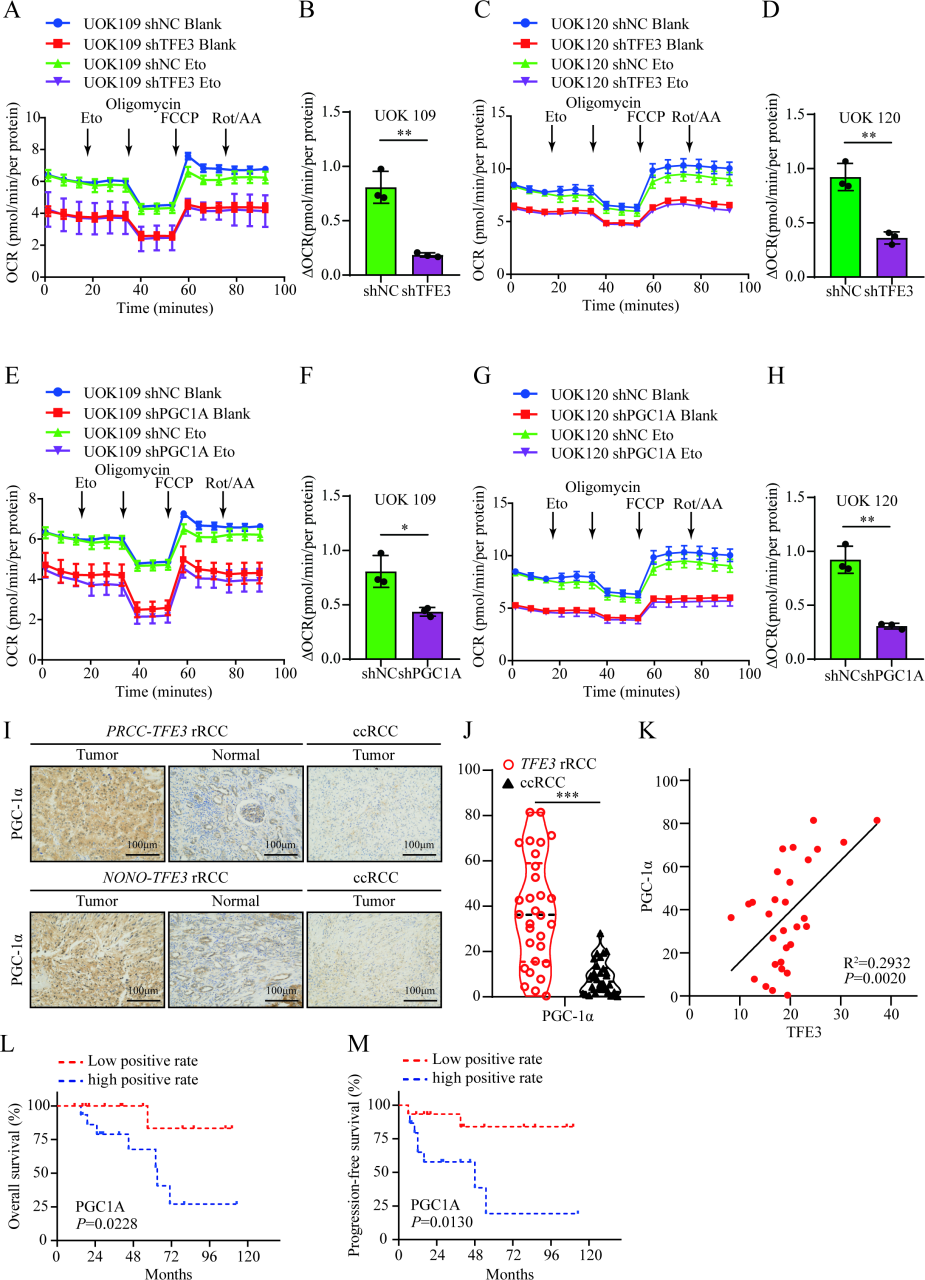
*TFE3* fusion proteins and PGC-1α enhance mitochondrial respiratory dependency on FAs in *TFE3* rRCC and influence patient prognosis. **(A–D)** Seahorse XF96 analysis of mitochondrial maximal respiratory response to long-chain FAO stress, with quantitative analysis of OCR response to the FAO inhibitor Eto during maximal mitochondrial respiration in UOK109 and UOK120 cells (shNC and sh*TFE3*). **(E–H)** Seahorse XF96 analysis of mitochondrial maximal respiratory response to long-chain FAO stress, with quantitative analysis of OCR response to the FAO inhibitor Eto during maximal mitochondrial respiration in UOK109 and UOK120 cells (shNC and sh*PGC1A*). **(I)** Immunohistochemical detection of PGC-1α expression in human *TFE3* rRCC and ccRCC tumor tissues. **(J)** Quantification of PGC-1α levels in human *TFE3* rRCC and ccRCC tumor tissues. **(K)** Quantification of the correlation between *TFE3* fusion proteins and PGC-1α expression levels. **(L)** Kaplan-Meier analysis comparing OS between low and high PGC-1α expression groups in *TFE3* rRCC patients. **(M)** Kaplan-Meier analysis comparing PFS between low and high LAMP2A expression groups in *TFE3* rRCC patients. *P < 0.05; **P < 0.01; ***P < 0.001.

Previous research has demonstrated that PGC-1α modulates the expression of mitochondrial FAO enzymes, thereby increasing the rate of mitochondrial FAO (19). We proposed that TFE3 fusion proteins may enhance the mitochondrial oxidation of FAs via upregulating PGC-1α. To test this hypothesis, the standard XF long-chain FAO stress assay kit was utilized to evaluate the extent to which mitochondrial maximal respiratory capacity in UOK109 and UOK120 cells depends on long-chain FAO, both with and without *TFE3* fusion gene knockdown. The results demonstrated that, compared to the control group, the experimental group (with *TFE3* fusion gene knockdown) showed a significantly reduced oxygen consumption rate (OCR) in response to the FAO inhibitor Eto during maximal mitochondrial respiration. This reduction suggested that interference with TFE3 fusion proteins significantly reduced the tumor cells’ reliance on long-chain FAO for maximal mitochondrial respiratory capacity (**Figures 4A–D**). A lentiviral transfection system was used to introduce shRNAs into UOK109 and UOK120 cells, establishing stable *PGC1A* knockdown cell models. Western blotting and qRT-PCR were performed to verify the downregulation of PGC1A protein and mRNA. The results showed that the shPGC1A lentivirus was highly effective compared to the control group (**Supplementary Figures S2A–C**). Subsequently, the standard XF long-chain FAO stress assay kit was employed to evaluate the dependency of mitochondrial maximal respiratory capacity on long-chain FAO before and after *PGC1A* knockdown in UOK109 and UOK120 cells. Seahorse analysis revealed that, compared to the control group, tumor cells with *PGC1A* knockdown showed a significantly reduced OCR in response to the FAO inhibitor Eto during maximal mitochondrial respiration. This indicated that silencing *PGC1A* significantly decreased the tumor cells’ reliance on long-chain FAO for maximal mitochondrial respiratory capacity (**Figures 4E–H**). To further clarify whether PGC-1α influenced the progression of *TFE3* rRCC, immunohistochemical staining was performed on human *TFE3* rRCC and ccRCC tumor tissue samples. The results revealed that PGC-1α expression was significantly higher in *TFE3* rRCC tumor tissues compared to ccRCC (**Figures 4I, J**). Quantitative analysis indicated a strong correlation between *TFE3* fusion proteins and PGC-1α expression in tumor tissues (**Figure 4K**). Using the median PGC-1α expression level in tumor tissues as a cutoff, patients were divided into low-positive and high-positive groups. Kaplan-Meier survival analysis demonstrated that patients in the high-positive PGC-1α expression group had significantly poorer OS and progression-free survival (PFS) compared to those in the low-positive group (**Figures 4L, M**). These findings suggested that high expression of PGC-1α promotes the progression of *TFE3* rRCC. To assess the biological impact of PGC-1α on *TFE3* rRCC cells, CCK-8 assays showed that knockdown of *PGC1A* significantly suppressed the proliferation of UOK109 and UOK120 cells (**Supplementary Figures S2D, E**). EdU assays revealed that *PGC1A* knockdown also notably inhibited DNA replication activity in both UOK109 and UOK120 cells (**Supplementary Figures S2F, H, I**). Likewise, clone formation assays demonstrated that silencing *PGC1A* significantly reduced the clonogenic potential of UOK109 and UOK120 cells (**Supplementary Figures S2G, J, K**). Apoptosis detection indicated a significant increase in apoptotic cells following *PGC1A* downregulation in both UOK109 and UOK120 cells (**Supplementary Figure S2L**). Transwell assays further showed that *PGC1A* knockdown resulted in a marked decrease in the number of cells migrating and invading the lower chamber in both UOK109 and UOK120 cells (**Supplementary Figures S2M–Q**).

To confirmed whether TFE3 fusion proteins promote *TFE3* rRCC progression through the increasing *PGC1A* expression, a series of rescue experiments were performed. Flag-tagged *PGC1A* was transfected into 786-O cells to establish a model of *PGC1A* overexpression. Western blotting and qRT-PCR confirmed the upregulation of *PGC1A* mRNA and protein, showing that the *PGC1A* overexpression virus was effective compared to the control group (**Supplementary Figures S3A, B**). When the *TFE3* fusion gene was knocked down in UOK109 and UOK120 cells, cell proliferation and clonogenic ability were significantly impaired. However, when *PGC1A* was upregulated simultaneously with *TFE3* fusion gene knockdown, the proliferation and clonogenic potential of the cells were largely restored to levels comparable to the control group (**Supplementary Figures S3C, D, H, J**). Similarly, EdU assays showed that DNA replication rates were significantly reduced upon *TFE3* fusion gene knockdown in UOK109 and UOK120 cells, while simultaneous upregulation of *PGC1A* alleviated the inhibition of DNA replication (**Supplementary Figures S3E–G**). Consistent with these findings, in 786-O cells, upregulation of the *TFE3* fusion gene coupled with downregulation of *PGC1A* resulted in significantly reduced cell proliferation, as shown by CCK-8 assays, and a marked decrease in DNA replication, as indicated by EdU assays (**Supplementary Figures S3K–N**). These results suggested that TFE3 fusion proteins enhanced cell proliferation, anti-apoptotic activity, and migration/invasion by promoting *PGC1A* expression, thus driving the progression of *TFE3* rRCC.

### 3.4 PGC-1α promotes FAO by co-activating PPARα to upregulate CPT1A

**Figure 5:**
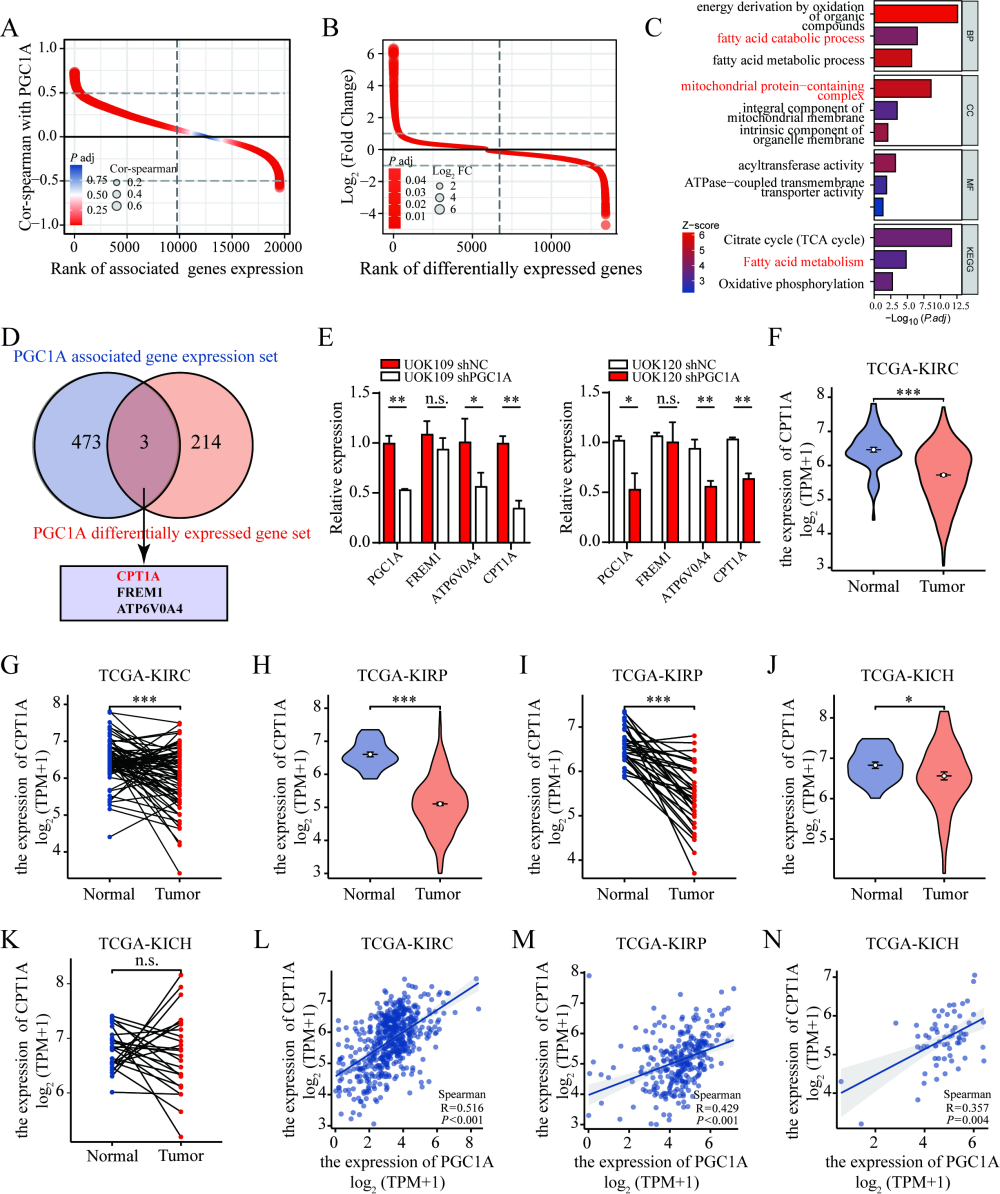
CPT1A as a potential downstream effector gene of *PGC1A*. **(A)** Differential ranking plot showing the gene set correlated with *PGC1A*, generated through single-gene correlation analysis. **(B)** Differential ranking plot displaying the gene set with differential *PGC1A* expression, identified by single-gene differential expression analysis. **(C)** GO and KEGG pathway analysis of the gene set derived from the *PGC1A* single-gene correlation analysis. **(D)** Venn diagram illustrating the overlap between the *PGC1A*-correlated gene set and the differentially expressed gene set. **(E)** Real-time PCR analysis of changes in CPT1A expression following *PGC1A* knockdown in UOK109 and UOK120 cells. **(F–K)** TCGA database analysis of differential expression levels of CPT1A between KIRC, KIRP, KICH, and normal tissues. **(L–N)** Correlation between CPT1A and *PGC1A* expression levels in tumor tissues from KIRC, KIRP, and KICH patients. *P < 0.05; **P < 0.01; ***P < 0.001.

To explore the impact of high PGC-1α expression in *TFE3* rRCC on mitochondrial FAs metabolism, bioinformatics analysis was performed using the KIRC dataset from the TCGA database. A single-gene correlation analysis based on the KIRC dataset was conducted to generate a gene set associated with *PGC1A* expression. The correlation values of all genes in this gene set are depicted in the ranking plot (**Figure 5A**). GO analysis of the gene set revealed that biological processes enriched in the *PGC1A*-associated gene set included mitochondrial protein synthesis, energy metabolism, palmitoyltransferase activity, and FAs catabolic processes. KEGG pathway analysis further indicated that the gene set was enriched in pathways related to the mitochondrial tricarboxylic acid cycle, FAs metabolism, and oxidative phosphorylation (**Figure 5C**). Similarly, differential gene expression analysis was performed based on the KIRC dataset to generate a gene set of DEGs related to *PGC1A*. The expression differences of these genes are shown in the ranking plot (**Figure 5B**). The intersection of genes from the *PGC1A*-associated gene set and the differential expression gene set, selecting genes with an absolute correlation value ≥ 0.5 and absolute Log2 fold change ≥2, resulted in three genes: *CPT1A, FREM1, and ATP6V0A4* (**Figure 5D**).

Subsequently, *PGC1A* was knocked down in UOK109 and UOK120 cells, and the expression levels of CPT1A, FREM1, and ATP6V0A4 were measured using qRT-PCR. The results showed that the expression levels of CPT1A and ATP6V0A4 were significantly reduced (**Figure 5E**). CPT1A, located on the outer mitochondrial membrane, is crucial for mitochondrial FAO (18). Using the TCGA database, expression levels of *CPT1A* in common RCC subtypes (KIRC, KIRP, and KICH) were analyzed. Compared to normal tissue, *CPT1A* expression was lower in KIRC, KIRP, and KICH tissues (**Figures 5F–K**). Correlation analysis from the TCGA database revealed that, in KIRC, KIRP, and KICH tumor tissues, CPT1A expression was significantly positively correlated with *PGC1A* expression (**Figures 5L, M**). These findings suggested that *CPT1A* may be a potential downstream effector gene of *PGC1A*.

**Figure 6:**
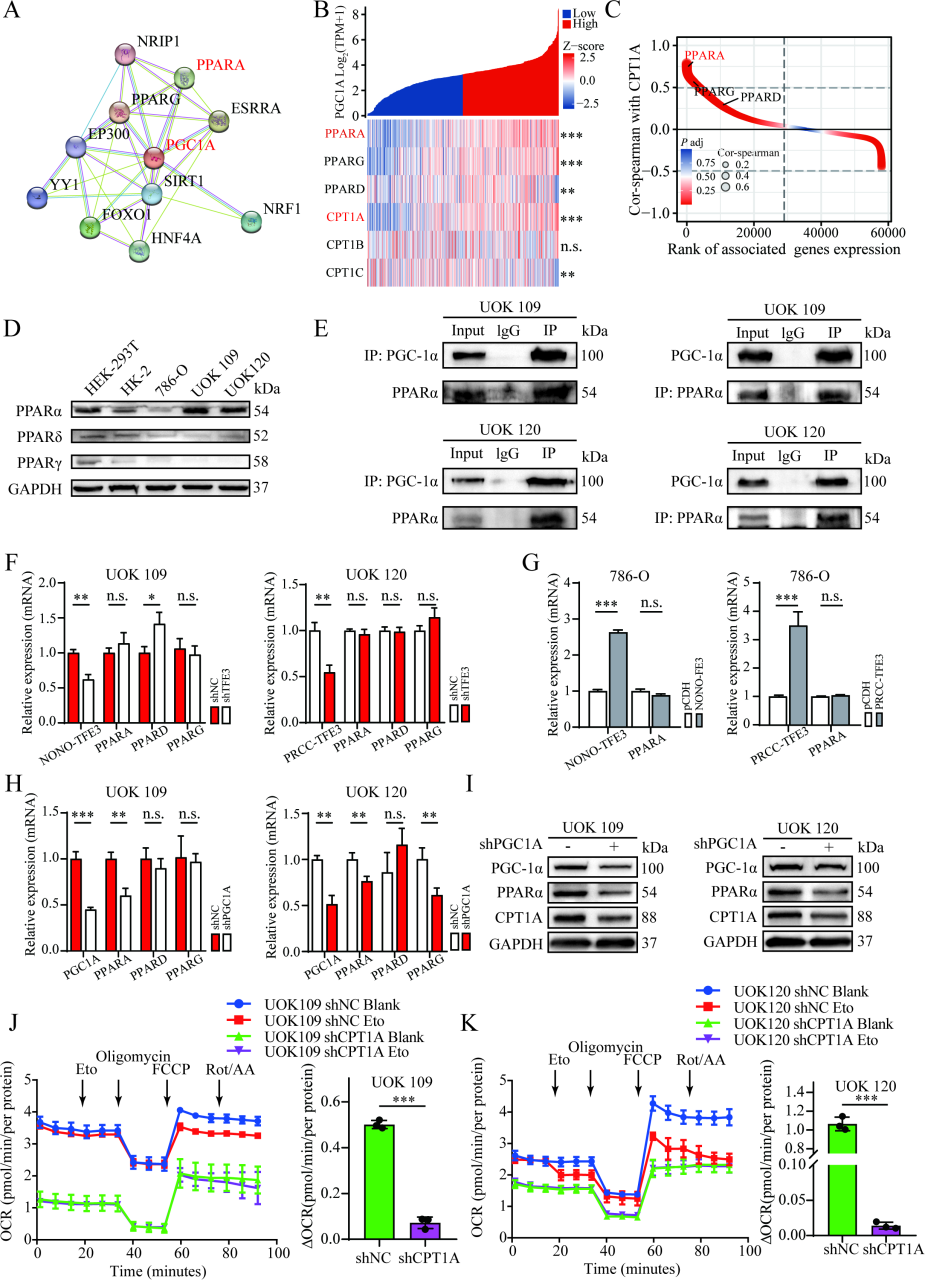
PGC-1α coactivates transcription factor PPARα to regulate CPT1A expression, enhancing mitochondrial dependency on FAO in *TFE3* rRCC. **(A)** Molecular interaction analysis of PGC-1α using the STRING database. **(B)** Co-expression heatmap showing the expression levels of *PGC1A*, *PPARA*, and *CPT1A*. **(C)** Differential ranking plot from single-gene correlation analysis showing the gene set correlated with CPT1A. **(D)** Western blot analysis of PPAR family protein expression in various cell lines. **(E)** Co-IP analysis confirming the interaction between PGC-1α and PPARα in UOK109 and UOK120 cells. **(F, G)** Real-time PCR analysis of PPARA expression levels following knockdown or overexpression of *TFE3* fusion genes. **(H, I)** Western blot and Real-time PCR analysis of PPARA and CPT1A protein and mRNA expression levels following *PGC1A* silencing. *P < 0.05; **P < 0.01; ***P < 0.001; n.s.: Not significant. **(J)** Seahorse XF96 analysis of mitochondrial maximal respiratory response to long-chain FAO stress, with quantitative analysis of OCR response to the FAO inhibitor Eto during maximal mitochondrial respiration in UOK109 shNC and shCPT1A cells. **(K)** Seahorse XF96 analysis of mitochondrial maximal respiratory response to long-chain FAO stress, with quantitative analysis of OCR response to Eto during maximal mitochondrial respiration in UOK120 shNC and shCPT1A cells. *P < 0.05; **P < 0.01; ***P < 0.001.

Previous studies have demonstrated that PGC-1α coactivates the transcription factor PPARα to regulate mitochondrial FAO enzymes. Based on this, it was hypothesized that this pathway could regulate the expression of *CPT1A* in *TFE3* rRCC. To test this hypothesis, potential molecular interactions with PGC-1α were predicted using the STRING database (**Figure 6A**). The results indicated that PGC-1α interacted with several transcription factors, including PPARα. Additionally, co-expression heatmap analysis revealed that as *PGC1A* expression increased, the expression levels of PPARα and CPT1A also showed a corresponding increase (**Figure 6B**). Correlation analysis of the KIRC dataset showed a significant positive correlation between *PPARα* and *CPT1A* expression levels (**Figure 6C**). Western blot analysis confirmed that PPARα protein levels were significantly elevated in UOK109 and UOK120 cells compared to normal renal tubular epithelial cells (HK-2) (**Figure 6D**). To verify the interaction between PGC-1α and PPARα in UOK109 and UOK120 cells, Co-IP experiments were performed using total cell lysates from these cell lines. The results demonstrated that PGC-1α and PPARα interact (**Figure 6E**). Knockdown or overexpression of the *TFE3* fusion gene did not affect PPARα expression levels (**Figures 6F, G**), but silencing *PGC1A* significantly decreased the mRNA and protein levels of both PPARα and CPT1A in tumor cells (**Figures 6H, I**).

**Figure 7:**
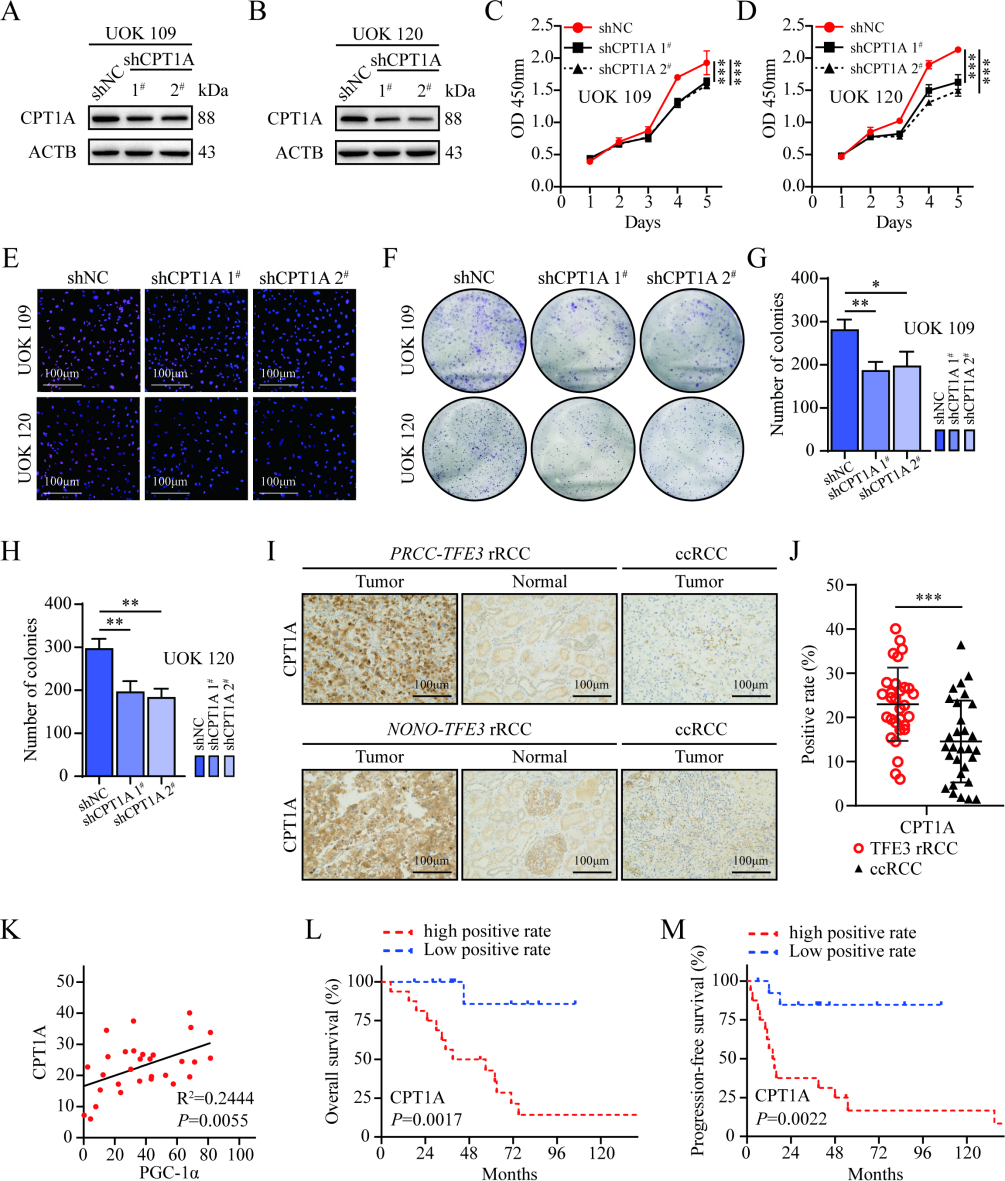
High expression of CPT1A promotes *TFE3* rRCC tumor progression. **(A, B)** Western blot analysis of CPT1A expression in UOK109 and UOK120 cells transfected with shCPT1A virus. **(C, D)** CCK-8 assays measuring the proliferation capacity of UOK109 and UOK120 cells transfected with the respective virus. **(E)** EdU assay to assess DNA replication in UOK109 and UOK120 cells transfected with the respective virus. **(F–H)** Clone formation assays evaluating the clonogenic potential of UOK109 and UOK120 cells transfected with the respective virus. **(I)** Immunohistochemical analysis of CPT1A expression in human *TFE3* rRCC and ccRCC tumor samples. **(J)** Quantification of CPT1A expression levels in human *TFE3* rRCC and ccRCC tumor samples. **(K)** Quantitative analysis of the correlation between CPT1A and PGC-1α expression levels. **(L)** Kaplan-Meier analysis comparing OS between low and high CPT1A expression groups in *TFE3* rRCC patients. **(M)** Kaplan-Meier analysis comparing PFS between low and high CPT1A expression groups in *TFE3* rRCC patients. *P < 0.05; **P < 0.01; ***P < 0.001.

To explore the role of CPT1A in the mitochondrial FAO process in *TFE3* rRCC cells, a lentiviral transfection system was used to introduce shNC and sh*CPT1A* shRNAs into UOK109 and UOK120 cells, establishing stable *CPT1A* knockdown cell models. Western blotting confirmed effective *CPT1A* knockdown using the sh*CPT1A* lentivirus (**Figures 7A, B**). The dependency of mitochondrial maximal respiratory capacity on long-chain FAO in these cells was then assessed before and after *CPT1A* knockdown using the standard XF long-chain FAO stress assay kit. Seahorse analysis showed that, compared to the control group, *CPT1A* knockdown significantly reduced the OCR in response to the FAO inhibitor Eto during maximal mitochondrial respiration, indicating that silencing *CPT1A* significantly reduces the tumor cells’ reliance on long-chain FAO for maximal mitochondrial respiratory capacity (**Figures 6J, K**). Taken together, these results demonstrated that PGC-1α interacted with PPARα to upregulate *CPT1A* expression, thereby promoting FAO in *TFE3* rRCC cells.

### 3.5 Knockdown of CPT1A inhibits *TFE3* rRCC progression

To evaluate the biological impact of CPT1A on *TFE3* rRCC cells, CCK-8 assays revealed that knockdown of *CPT1A* markedly inhibited the proliferation of UOK109 and UOK120 cells (**Figures 7C, D**). EdU assays also showed that *CPT1A* knockdown significantly suppressed DNA replication activity in these cells (**Figure 7E**). Likewise, clone formation assays demonstrated that silencing CPT1A significantly reduced the clonogenic potential of UOK109 and UOK120 cells (**Figures 7F, H**).

Immunohistochemical staining of human *TFE3* rRCC and ccRCC tumor tissue samples revealed significantly higher CPT1A expression in *TFE3* rRCC tissues compared to ccRCC, consistent with the cellular experimental results (**Figures 7I, J**). Quantitative analysis showed a strong positive correlation between CPT1A and PGC-1α expression levels in tumor tissues (**Figure 7K**). Using the median CPT1A expression level in tumor tissues as a cutoff, patients were divided into low-positive and high-positive groups. Kaplan-Meier survival analysis revealed that patients in the high-positive CPT1A group had significantly worse OS and PFS compared to those in the low-positive group (**Figures 7L, M**).

**Figure 8:**
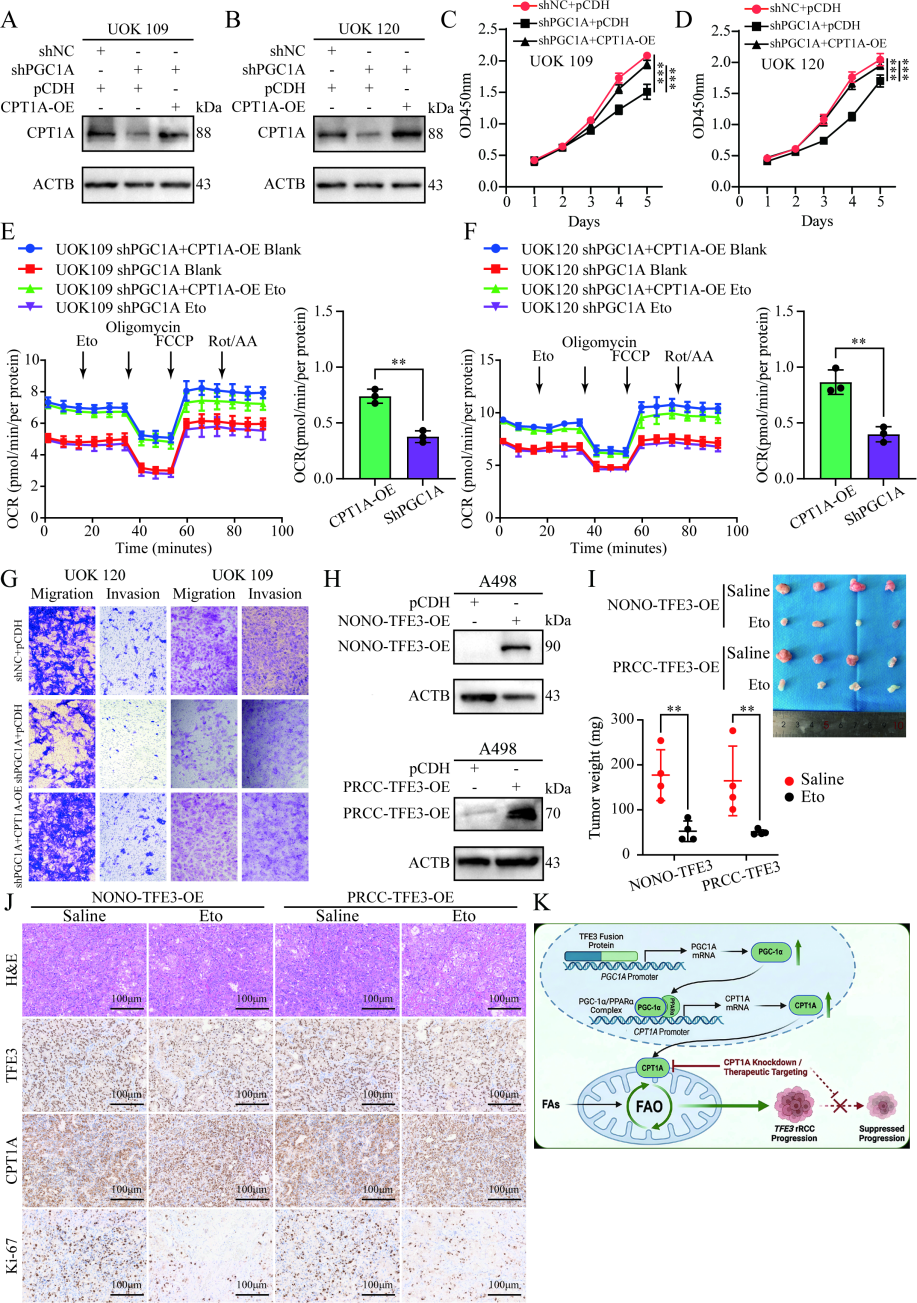
PGC1α promotes the progression of *TFE3* rRCC via CPT1A, and the CPT1A inhibitor etomoxir can suppress tumor growth *in vivo*. **(A, B)** Western blot analysis of the knockdown and overexpression efficiency of PGC1α and CPT1A in UOK109 and UOK120 cells. **(C, D)** CCK-8 assays to measure the proliferation capacity of UOK109 and UOK120 cells in each group. **(E, F)** Seahorse XF96 analysis of mitochondrial maximal respiratory response to long-chain FAO stress, with quantitative analysis of OCR response to the FAO inhibitor Eto during maximal mitochondrial respiration in UOK109 and UOK120 cells in each group. **(G)** Transwell assays to evaluate the migration and invasion abilities of UOK109 and UOK120 cells in each group. **(H)** Western blot analysis of the overexpression efficiency of NONO-TFE3 and PRCC-TFE3 in A498 cells. **(I)** Subcutaneous tumor xenograft models in nude mice were established using A498 cells overexpressing *NONO-TFE3* or *PRCC-TFE3*. The volume and weight of tumors formed in nude mice injected with Eto or normal saline were compared. **(J)** Partial tumor sections were subjected to H&E staining and IHC staining for TFE3, CPT1A, and Ki-67. **(K)** The mechanism by which chimeric TFE3 fusion proteins influence tumor progression in *TFE3* rRCC by regulating mitochondrial FAO. *P < 0.05; **P < 0.01; ***P < 0.001.

To further verify the core mechanism by which PGC1α regulates *TFE3* rRCC progression through CPT1A, we performed a series of rescue experiments by knocking down *PGC1A* followed by overexpressing *CPT1A* in UOK109 and UOK120 cells (**Figures 8A, B**). Results from CCK-8 proliferation assays, Seahorse XF96 long-chain FAO stress tests, and Transwell migration and invasion assays showed that knockdown of *PGC1A* in UOK109 and UOK120 cells led to reduced cell proliferation, decreased mitochondrial dependency on long-chain FAO, and impaired cell migration and invasion capabilities. In contrast, overexpression of *CPT1A* in *PGC1A*-knockdown cells largely restored the aforementioned phenotypes to the levels of the control group (**Figures 8C–G**, **Supplementary Figures S3O, P**). These results clearly confirm that PGC1α influences lipid metabolism and thereby regulates *TFE3* rRCC progression through CPT1A.

Given the poor tumorigenicity of patient-derived TFE3 rRCC cell lines in immunocompromised mice, we established xenograft models using A498 cells stably overexpressing NONO-TFE3 and PRCC-TFE3 to evaluate *in vivo* efficacy (**Figures 8H, J**). To observe the tumor response to the CPT1A inhibitor Eto, we began intraperitoneal injection of Eto daily in mice 10 days after subcutaneous tumor implantation, and tumors were harvested after 3 weeks of injection. The results showed that Eto significantly inhibited tumor growth (**Figure 8I**). Immunohistochemical staining further confirmed that Eto administration did not affect the expression of CPT1A, but its inhibitory effect on CPT1A functional activity significantly reduced the proliferation capacity of tumor cells, as indicated by the Ki-67 positive rate (**Figure 8J**). These findings suggested that high expression of CPT1A promotes the progression of *TFE3* rRCC (**Figure 8K**).

## 4 Discussion

A hallmark of malignant tumors is the reprogramming of metabolic processes to meet the energy and material demands necessary for tumor cell growth, invasion, and metastasis. FAs can be sourced not only from the extracellular environment but also from intracellular lipid droplets (LDs) via the process of lipophagy (21, 22). Our previous work demonstrated that *TFE3* rRCC exhibits relatively low LDs accumulation due to TFE3 fusion proteins inhibiting LD biosynthesis and enhancing LD degradation via chaperone-mediated lipophagy (CMA) through LAMP2A upregulation (13). This study extends our understanding by revealing that TFE3 fusion proteins drive tumor progression not only through enhanced LD degradation but also by transcriptionally upregulating *PGC1A* to promote mitochondrial FAO, providing a robust energy source for aggressive tumor behavior. The degradation of LDs via lipophagy would release FAs, which can then be utilized as substrates for the enhanced FAO pathway, suggesting a coordinated metabolic strategy in *TFE3* rRCC.

PGC-1α is a key regulator of mitochondrial biogenesis and metabolism across various malignant tumors, playing a crucial role in tumor progression (23, 24). Its role, however, can be context-dependent. For example, in malignant melanoma stem cells, elevated PGC-1α expression significantly enhances the biological functions of tumor stem cells, and inhibiting its expression can markedly suppress these functions (25). Similarly, in liver cancer cells, low PGC-1α expression leads to lipid accumulation, promoting tumor progression (26). Conversely, certain studies suggest that PGC-1α may function as a tumor suppressor in prostate cancer, where its reduced expression accelerates tumor growth (27). These varied findings highlight that PGC-1α expression levels and its functional role vary across different malignancies.

In this study, we observed a complex pattern regarding PGC-1α expression and patient prognosis. Our bioinformatics analysis of general kidney cancers (KIRC and KIRP) from the TCGA database indicated that low *PGC1A* expression correlated with poorer prognosis. This aligns with some literature suggesting a tumor-suppressive role or that loss of metabolic efficiency can be detrimental. However, in our specific cohort of *TFE3* rRCC patients, high expression of PGC-1α was observed in tumor tissues and correlated with poor patient prognosis, indicating an oncogenic role for PGC-1α unique to this subtype. This apparent discrepancy underscores the heterogeneity of kidney cancers and highlights the distinct metabolic adaptations of *TFE3* rRCC. Unlike other RCC subtypes where PGC-1α might play a more suppressive or context-dependent role, in *TFE3* rRCC, its upregulation by fusion proteins appears to specifically fuel aggressive tumor characteristics, leading to worse outcomes. Given the development of selective PGC-1α inhibitors, further research is warranted to explore the therapeutic potential of these inhibitors in suppressing *TFE3* rRCC progression, particularly considering its context-specific oncogenic role.

CPT1A, the rate-limiting enzyme in mitochondrial FAO, is frequently highly expressed in various tumors, providing essential energy for tumor cell survival and growth. For example, in hepatocellular carcinoma, elevated CPT1A expression promotes FAO and breakdown, contributing to malignancy (28). Conversely, inhibiting CPT1A in glioblastoma significantly reduces tumor cell viability and invasiveness (29). Interestingly, in ccRCC tumor tissues, CPT1A has been reported to reduce lipid accumulation in tumor cells and inhibit cell proliferation (30–32), again highlighting context-specific roles. In the present study, CPT1A was found to be highly expressed in *TFE3* rRCC tumor tissues, and crucially, this high expression correlated with poor patient prognosis. Mechanistically, these findings suggest that PGC-1α regulates PPARα/CPT1A axis to promote tumor progression while also enhancing the mitochondrial dependency on long-chain FAO in *TFE3* rRCC cells. Although differential gene pathway analysis from some *TFE3* rRCC proteomics data has revealed suppression of the FA catabolic pathway, this might stem from the tumor heterogeneity or reflect specific aspects of FA metabolism that differ from FAO. Our data strongly supports FAO as a key metabolic driver in *TFE3* rRCC.

FA metabolism is intricately regulated by both mitochondrial oxidation and intracellular synthesis enzymes. While lipid metabolism abnormalities in ccRCC have been extensively studied (33–37), *TFE3* rRCC, with its unique genetic background, exhibits distinct metabolic characteristics (5, 38, 39). This study demonstrates that TFE3 fusion proteins enhance mitochondrial FAO through the PGC-1α/PPARα/CPT1A axis, thereby promoting tumor progression. These findings provide new insights into potential therapeutic targets for *TFE3* rRCC, particularly by disrupting its reliance on FAO.

Regrettably, the absence of *in vivo* animal experiments limits the translational potential of our findings, as physiological and pathological responses in a complex biological system cannot be fully replicated *in vitro*. We will continue our efforts to isolate and extract primary cells and conduct animal experiments to further validate and expand our current research findings.

## 5 Conclusion

This study revealed that *PGC1A is* a potential downstream target of TFE3 fusion proteins and its expression correlated with *TFE3* rRCC prognosis. We demonstrated that TFE3 fusion proteins transcriptionally upregulated *PGC1A* without altering its subcellular localization. Elevated PGC-1α, in turn, enhanced FAO and drove *TFE3* rRCC progression by co-activating PPARα to upregulate *CPT1A*. Critically, knockdown of *CPT1A* suppressed *TFE3* rRCC progression, highlighting this pathway as a promising therapeutic target. Our findings shed light on the unique metabolic reprogramming driven by TFE3 fusion proteins in *TFE3* rRCC, offering a rationale for developing FAO-targeting therapies.

## Data Availability

The original contributions presented in the study are included in the article/**Supplementary Material**. Further inquiries can be directed to the corresponding author.

## Glossary

*TFE3* rRCC: *TFE3* rearranged renal cell carcinoma
RCC: renal cell carcinoma
PGC1A: peroxisome proliferator-activated receptor γ coactivator 1 alpha
PPARs: peroxisome proliferator-activated receptors
Fas: fatty acids
CPT1: Carnitine palmitoyltransferase 1
TCGA: The Cancer Genome Atlas
GEO: Gene Expression Omnibus
KIRC: Kidney Renal Clear Cell Carcinoma
KIRP: Kidney Renal Papillary Cell Carcinoma
KICH: Kidney Chromophobe
DEGs: differentially expressed genes
GO: Gene Ontology
KEGG: Genes and Genomes
OS: overall survival
DSS: disease-specific survival
PFI: progression-free interval
ROC: receiver operating characteristic
AUC: the area under the curve
IHC: immunohistochemistry
ccRCC: clear cell renal cell carcinoma
DMEM: Dulbecco's Modified Eagle Medium
FBS: fetal bovine serum
Co-IP: Co-Immunoprecipitation
IF: Immunofluorescence
FAO: fatty acid oxidation
Eto: Etomoxi
FCCP: Carbonyl cyanide p-trifluoromethoxyphenylhydrazone
Rot/AA: Rotenone/Antimycin A
ChIP: Chromatin Immunoprecipitation
OCR: oxygen consumption rate
