# Supplementary material for: TFE3 fusion proteins drive TFE3 rearranged renal cell carcinoma progression via PGC-1α-mediated fatty acid oxidation
Source: Front Immunol. 2026 Feb 4;17:1700983. doi: 10.3389/fimmu.2026.1700983 (PMC12913416; doi:10.3389/fimmu.2026.1700983)
Supplement: Supplementary file 1 [file DataSheet1.pdf]

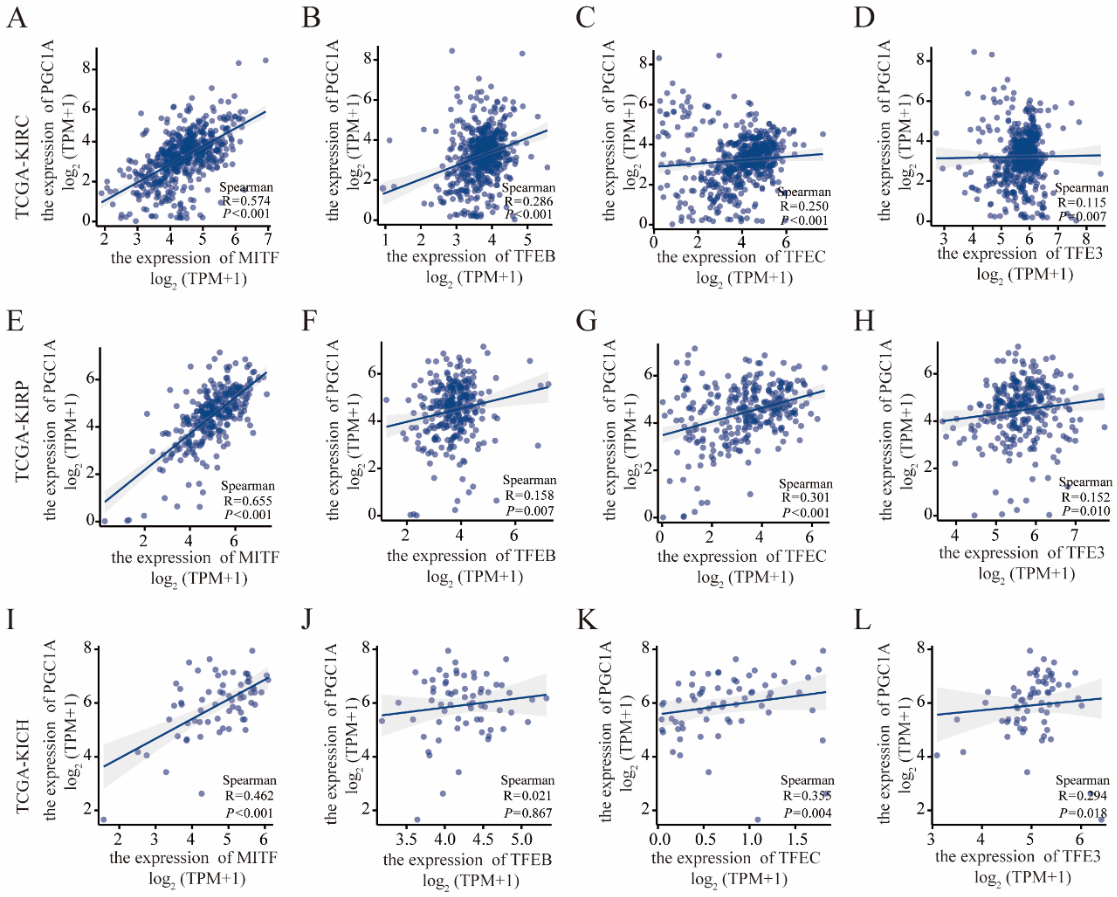

Figure S1 Correlation Between MiT Transcription Factor Family and *PGC1A* Expression. (A-D) Correlation analysis between MiT transcription factor family members and *PGC1A* expression in KIRC tumor tissues. (E-H) Correlation analysis between MiT transcription factor family members and *PGC1A* expression in KIRP tumor tissues. (I-L) Correlation analysis between MiT transcription factor family members and *PGC1A* expression in KICH tumor tissues. *P < 0.05; **P < 0.01; ***P < 0.001.

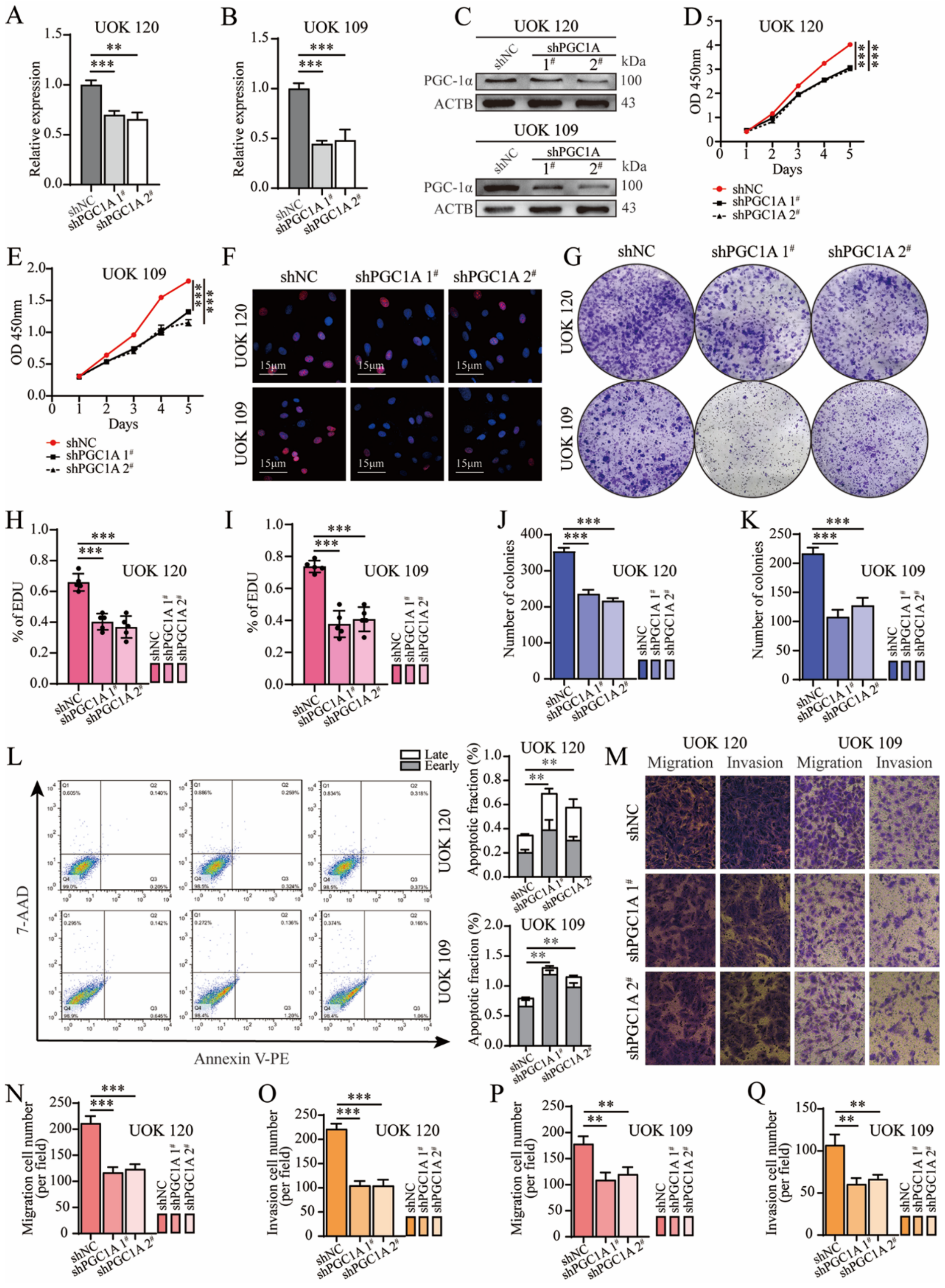

Figure S2. Silencing *PGC1A* Suppresses *TFE3* rRCC Tumor Progression. (A-C) Western blot and Real-time PCR analysis of *PGC1A* protein and mRNA levels in cells transfected with the respective virus. (D-E) CCK-8 assay assessing the proliferation of UOK109 and UOK120 cells after *PGC1A* knockdown. (F, H-I) EdU assay evaluating

DNA replication in UOK109 and UOK120 cells following *PGC1A* knockdown. (G, J-K) Clone formation assay measuring the clonogenic potential of UOK109 and UOK120 cells after *PGC1A* knockdown. (L) Flow cytometry analysis of apoptosis in UOK109 and UOK120 cells following *PGC1A* knockdown. (M-Q) Transwell assays assessing migration and invasion capabilities of UOK109 and UOK120 cells after *PGC1A* knockdown. *P < 0.05; **P < 0.01; ***P < 0.001.

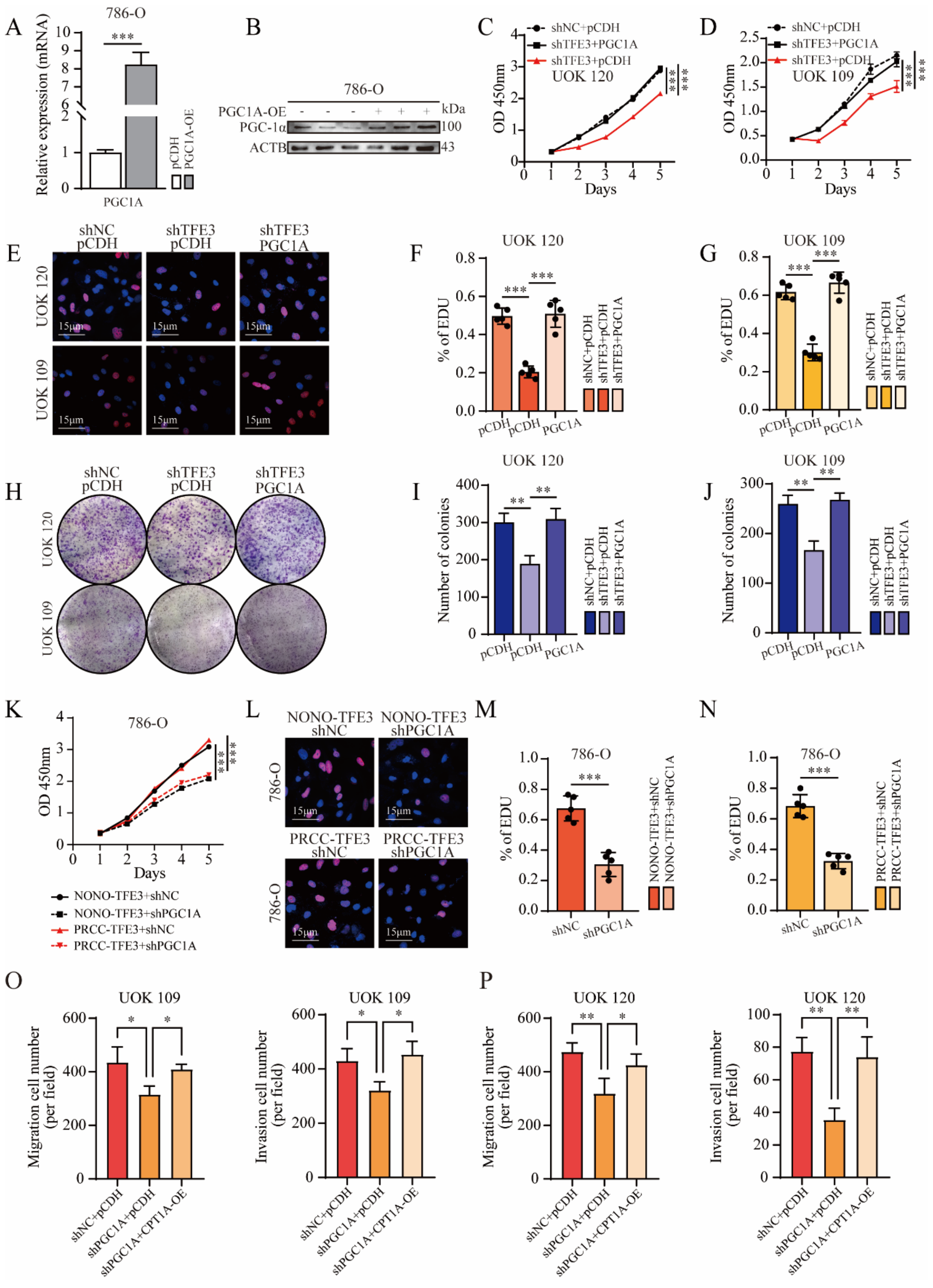

Figure S3. *TFE3* Fusion Proteins Upregulate PGC-1α to Promote *TFE3* rRCC Tumor Progression. (A-B) Western blot and Real-time PCR analysis of *PGC1A* protein and mRNA levels in cells transfected with the PGC-1α overexpression virus. (C-D) CCK-8 assay measuring the proliferation of UOK109 and UOK120 cells

transfected with the respective virus. (E-G) EdU assay assessing DNA replication in UOK109 and UOK120 cells transfected with the respective virus. (H-J) Clone formation assay evaluating the clonogenic potential of UOK109 and UOK120 cells transfected with the respective virus. (K) CCK-8 assay measuring the proliferation of 786-O cells transfected with the respective virus. (L-N) EdU assay evaluating DNA replication in 786-O cells transfected with the respective virus. (O-P) The Transwell migration and invasion assays to compare the number of transmembrane cells among different groups of UOK109 and UOK120 cells. $^{*}P < 0.05$; $^{**}P < 0.01$; $^{***}P < 0.001$.
